# The Use of 3D Printing Filaments to Build Moisture Sensors in Porous Materials

**DOI:** 10.3390/ma18010115

**Published:** 2024-12-30

**Authors:** Magdalena Paśnikowska-Łukaszuk, Joanna Szulżyk-Cieplak, Magda Wlazło, Jarosław Zubrzycki, Ewa Łazuka, Arkadiusz Urzędowski, Zbigniew Suchorab

**Affiliations:** 1 Faculty of Mathematics and Information Technology, Lublin University of Technology, Nadbystrzycka 38, 20-618 Lublin, Poland; m.pasnikowska-lukaszuk@pollub.pl (M.P.-Ł.); j.szulzyk-cieplak@pollub.pl (J.S.-C.); m.wlazlo@pollub.pl (M.W.); e.lazuka@pollub.pl (E.Ł.); a.urzedowski@pollub.pl (A.U.); 2 Faculty of Environmental Engineering, Lublin University of Technology, Nadbystrzycka 40B, 20-618 Lublin, Poland; z.suchorab@pollub.pl

**Keywords:** measurement methods, building materials, moisture transport, TDR, 3D printing, PLA

## Abstract

This study explores the application of materials used in 3D printing to manufacture the housings of non-invasive sensors employed in measurements using a TDR (Time Domain Reflectometry) meter. The research investigates whether sensors designed with 3D printing technology can serve as viable alternatives to conventional invasive and non-invasive sensors. This study focuses on innovative approaches to designing humidity sensors, utilizing Fused Deposition Modeling (FDM) technology to create housings for non-invasive sensors compatible with TDR devices. The paper discusses the use of 3D modeling technology in sensor design, with particular emphasis on materials used in 3D printing, notably polylactic acid (PLA). Environmental factors, such as moisture in building materials, are characterized, and the need for dedicated sensor designs is highlighted. The software utilized in the 3D modeling and printing processes is also described. The Materials and Methods Section provides a detailed account of the construction process for the non-invasive sensor housing and the preparation for moisture measurement in silicate materials using the designed sensor. A prototype sensor was successfully fabricated through 3D printing. Using the designed sensor, measurements were conducted on silicate samples soaked in aqueous solutions with water absorption levels ranging from 0% to 10%. Experimental validation involved testing silicate samples with the prototype sensor to evaluate its effectiveness. The electrical permittivity of the material was calculated, and the root-mean-square error (RMSE) was determined using classical computational methods and machine learning techniques. The RMSE obtained using the classical method was 0.70. The results obtained were further analyzed using machine learning models, including Gaussian Process Regression (GPR) and Support Vector Machine (SVM). The GPR model achieved an RMSE of 0.15, while the SVM model yielded an RMSE of 0.25. These findings confirm the sensor’s effectiveness and its potential for further research and practical applications.

## 1. Introduction

Three-dimensional printing is currently the fastest additive manufacturing technology, and widely utilized across virtually all fields of life and science [1,2]. The broad availability of materials facilitates its application in a diverse range of industries [3,4]. Currently, the most popular 3D printing method is FDM (Fused Deposition Modeling) [5]. This method involves the deposition molten material [6,7], applied layer by layer through a nozzle, onto the machine platform [8,9]. Each layer represents a cross-section of the printed detail [10]. This method is a powerful tool for producing complex components due to its adaptability, low cost, availability, and rapid prototyping time [11,12,13]. These features make it particularly suitable for applications such as the production of sensors, probe housings, and other components essential to research processes [14,15]. The most commonly used materials in 3D printing include PLA and ABS [16]. PLA (polylactide) is a biodegradable polymer that is classified as belonging to the group of aliphatic polyesters [17,18]. Corn starch and sugar cane are used to produce it. [3,19,20]. In addition to its use in FDM technology, PLA is widely employed in the production of disposable packaging [21]. Due to its biocompatibility and its degradation time of several years, PLA finds applications in medical fields, including in scaffolds, components of absorbable surgical sutures, bandages, stents, orthopedic implants, and tissue scaffolds [22,23,24]. Furthermore, PLA is recyclable material [25]. While considered biodegradable, PLA requires specific conditions, such as industrial composting, for efficient decomposition, and it can take a significant amount of time to fully degrade under natural conditions [26]. PLA melts at a temperature range of 185–210 °C [17,27]. While PLA degrades under invasive weather and environmental conditions, it maintains its properties for an extended period if stored appropriately [28]. ABS is another material frequently used in 3D printing, particularly for spare parts, and is similarly popular in FDM technology [29]. ABS, or acrylonitrile-butadiene-styrene, is a terpolymer classified as a polyplastic [30]. It is synthesized through the polymerization of 1,3-butadiene and the copolymerization of acrylonitrile with styrene, accompanied by the grafting of the resulting copolymer onto polybutadiene [13,31]. ABS is highly resistant to mechanical damage but requires a higher printing temperature compared to PLA [12,32]. Both PLA and ABS are relatively low-cost materials; due to PLA’s market availability and resistance to high humidity, it is an excellent choice for creating research tools such as probes or components of measuring devices [7,33]. This study explored the use of PLA to fabricate surface sensors for use with a Time Domain Reflectometry (TDR) meter. TDR is an electrical technique based on the principle of the reflectometric measurement of the dielectric properties of a medium [34,35,36,37]. It is traditionally an invasive measurement method, requiring probes to be inserted directly into a porous medium. However, ongoing research has increasingly focused on developing non-invasive measurement methods [38]. TDR measurement technology is widely used for assessing moisture levels in building partitions [39,40,41]. The issue of moisture in building partitions is highly relevant, directly affecting the performance of buildings and significantly influencing the internal environment [42]. Consequently, the development of advanced moisture detection techniques for partitions, as well as the creation of new measurement methods and the optimization of existing ones, plays a critical role. In this context, integrating replaceable components such as housings fabricated using 3D printing technology may accelerate research efforts [14]. Three-dimensional printing technology enables the production of various types of surface sensors tailored to the shape of the material being tested and the degree of moisture present. Importantly, the sensor housing itself is not significantly exposed to moisture, as it operates only in direct contact with the tested material. Moisture in building partitions remains one of the most pressing issues in the construction industry [35,38]. This phenomenon is particularly prevalent in the temperate climate of Poland [42,43]. It results from the typical functioning of buildings constructed using traditional technology, taking into account the transport of water released inside the rooms to the outside through the porous structure of building materials [44]. The primary factor contributing to moisture formation in building walls is water, which may originate from various sources [45]. Water in building partitions may occur as chemically bound water, free steam, or free water [46]. Additionally, it can manifest as sorption moisture [47]. Capillary water adversely affects the thermal properties of buildings, while condensation water contributes to the development of moisture [48]. In building partitions, this can lead to sick building syndrome and the growth of fungi harmful to human health [49]. To evaluate the application of 3D printing in the TDR measurement method, sensors fabricated using PLA were developed. A measurement methodology utilizing proprietary sensors was proposed, enabling the rapid and non-invasive assessment of the moisture content in silicate materials, commonly used in construction, under laboratory conditions. The research hypothesis was put forward that sensors designed using 3D technology can be used to measure environmental parameters using TDR.

## 2. Materials and Methods

### 2.1. Preparation of a 3D Model

**Figure 1:**
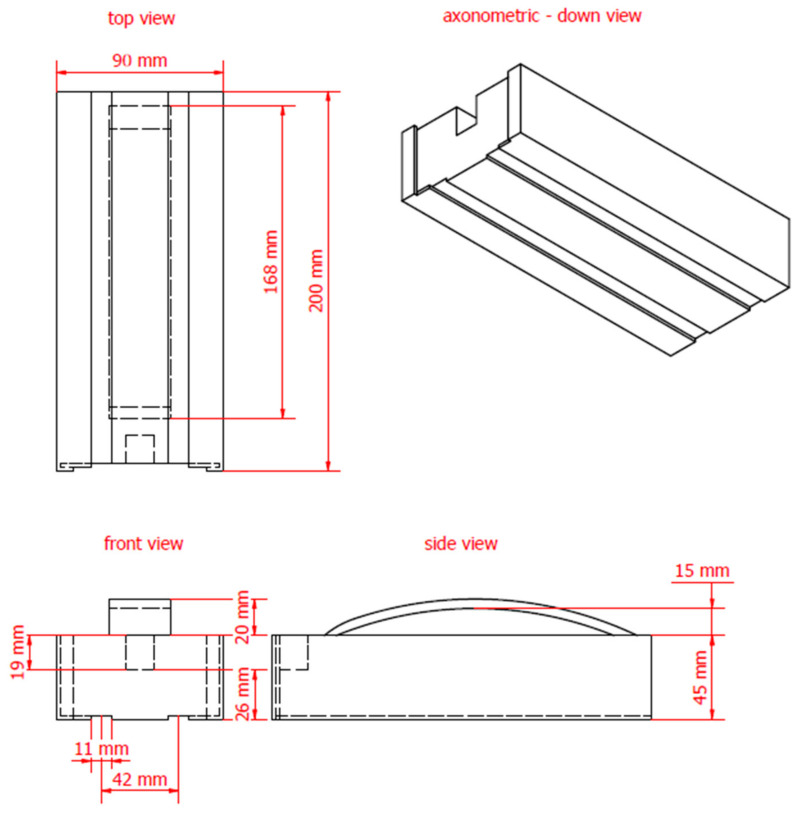
Dimensions of non-invasive 3D sensor.

The 3D model of the non-invasive surface sensor was designed using Autodesk Inventor, a software application specifically utilized for sketching and creating 3D models. It can also support engineering calculations, including strength analysis via the finite element method [50]. The structure and dimensions of the model are presented in Figure 1.

**Figure 2:**
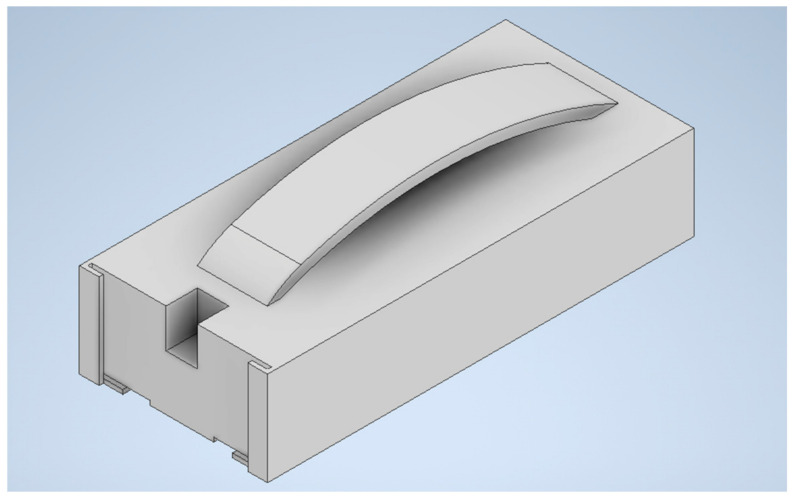
A 3D model of a non-invasive 3D sensor.

Subsequently, a 3D model was developed using modeling tools, and an .stl file was generated for 3D printing. A solid with the dimensions shown in Figure 1 was prepared. The .stl file was processed in Creality Slicer 4.8 to prepare a file with g.code extension. The model was configured for 100% fill. The 3D model is illustrated in Figure 2.

### 2.2. 3D Printing of the Sensor Housing

The sensor housing model was manufactured using an Ender 3 V2 printer using Fused Deposition Modeling (FDM) technology. This technique involves extruding material from the printer’s nozzle to build successive layers of the model by depositing molten material according to pre-calculated printing paths (g.code) [8,51,52]. The parameters of the 3D printer used for the sensor prototype are detailed in Table 1.

### 2.3. Sensor Housing Material

The sensor housing was printed using Deep Black PLA filament. The material parameters are summarized in Table 2. The apparent permittivity of the analyzed polylactide (PLA filament) is notably low, measured at 2.12 [14]. PLA is characterized by its insulating properties as it does not conduct electricity. Its chemical structure makes it an effective electrical insulator.

The parts manufactured using additive methods, such as 3D printing (in this case the FDM method), have similar strength parameters to those manufactured using classical methods (injection, casting, etc.) [53]. Strength tests conducted in the work of Zubrzycki et al. [53] on standardized samples for static tensile tests show the tensile strength of samples printed from PLA at different printing parameters (filling method—honeycomb, grid and line; different filling levels—10%, 30% and 60%). In these studies, a number of results were obtained, which showed that the Young’s modulus E*t* varied within the range from 1492.4 MPa for the sample printed with grid 10% filling to 2168.8 for the sample with 60% honeycomb. For the samples with 60% line filling, the E*t* was 2058 MPa. For the cited samples, the tensile strength σ*_m_* values were 28.31, 41.69, and 40.15 MPa, respectively. The percentage elongation ε*_m_* reached the values: 2.53, 2.54, and 2.5%. In turn, the tensile strength on failure σ*_b_* reached the values 28.31, 41.58, and 39.99 MPa.

The research shows that the destruction process of samples was different. The type of sample crack changed with the type of filling and the degree of the filling. 

To evaluate the water absorbability of samples that were 3D-printed from PLA material in the context of its application with materials exposed to aqueous solutions, the samples were subjected to immersion in a water bath. Five PLA samples, each with dimensions of 10 mm × 20 mm × 50 mm, were prepared using 3D printing technology. PLA samples were immersed in an aqueous solution with pH 7 and periodically weighed at several-day intervals to assess water absorption. Chemically pure water with a pH of 7 was chosen, verified using pH indicator papers. This pH level was selected to avoid interference from potential salts that could affect the measurements.

Following the measurements, the samples were stored in the same aqueous solution for one month in a tightly sealed environment shielded from light. The measurement results are presented in Table 3. Based on these findings, it can be concluded that short-term contact between the tested wet material and the sensor housed in a PLA casing does not significantly influence the process of measuring environmental parameters. Over such a short duration, the PLA casing does not absorb moisture from the tested building material.

### 2.4. Structural Components of the Sensor

**Figure 3:**
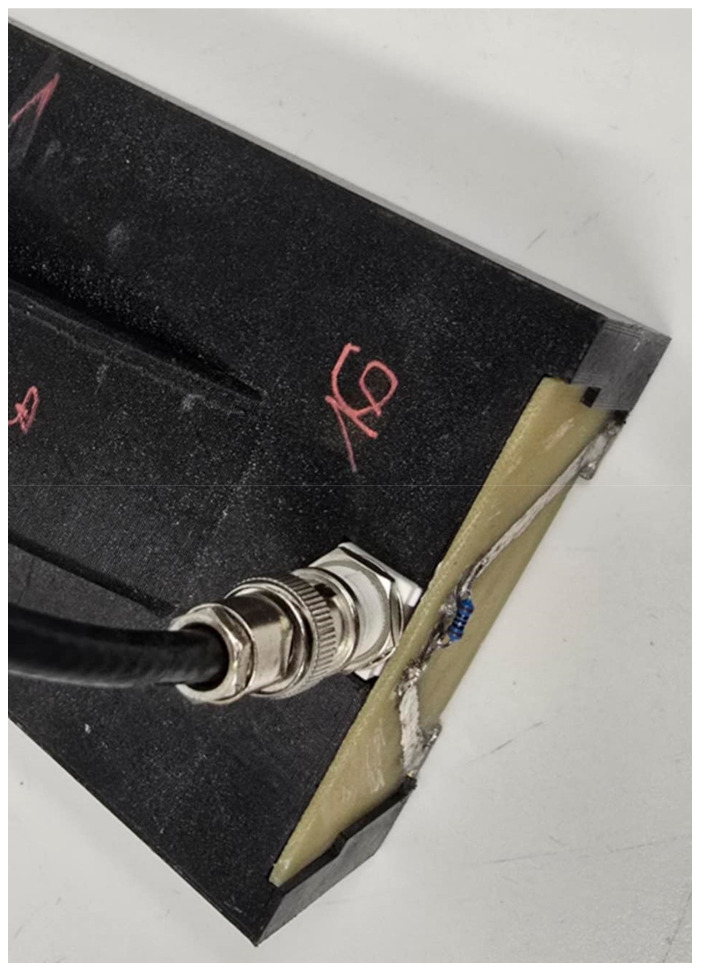
A view of the structural components of the sensor, including the printed circuit board, the BNC connector, and the attached coaxial cable.

The 3D sensor housing measures 200 mm in length. The spacing between the measurement rods, made of brass flat bars, is 42 mm. The waveguides, also constructed from brass, have dimensions of 10 mm × 2 mm × 200 mm. They are press-fitted into the 3D-printed housing. The flat bar is directly embedded in the housing, ensuring that the rods are solely in contact with the material under investigation. The probe communicates with the TDR meter via a BNC connector, which is soldered to a printed circuit board (PCB). The PCB connects the measurement rods to the connector. A coaxial cable is used to connect the probe to the TDR meter (Figure 3).

### 2.5. 3D Non-Invasive Sensor

**Figure 4:**
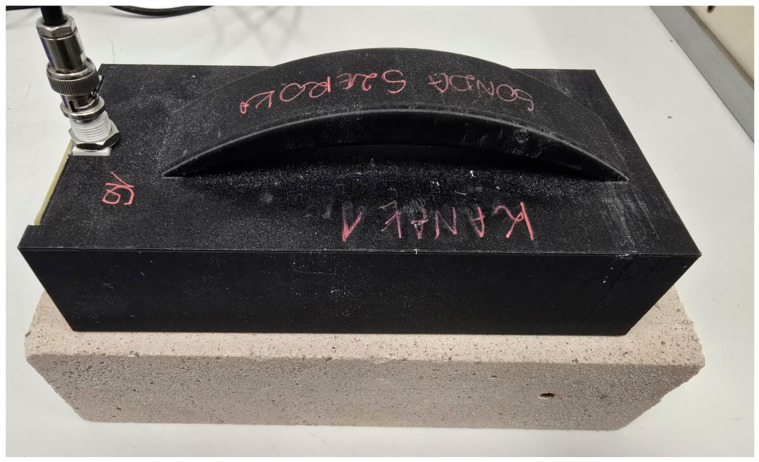
Sensor during tests on a silicate sample.

**Figure 5:**
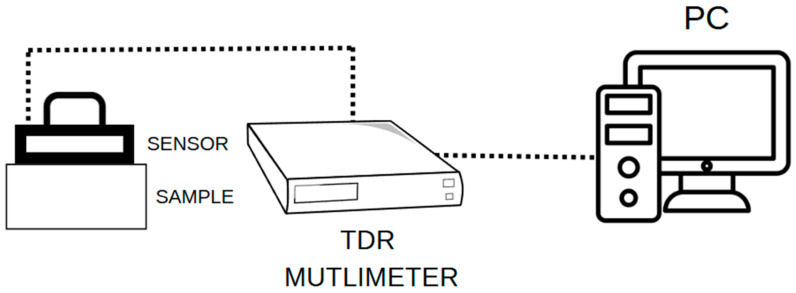
Diagram of the devices that were used during the tests.

The 3D-printed component serves as a housing for small electronics. The printed sensor housing incorporates a printed circuit board with a soldered angular BNC connector, which connects to 2 *×* 10 mm brass rods fabricated from flat bars. This configuration enables communication between the sensor and the TDR meter. The PLA housing does not interfere with measurement, as it acts as a dielectric component for the brass flat bars. The brass measuring rods are only in direct contact with the material being tested. Figure 4 llustrates the final configuration of the non-invasive moisture sensor, while Figure 5 presents a schematic of the devices that were used during the testing process.

### 2.6. Materials for Measurements

To conduct the tests, five silicate samples were prepared. The following equipment was utilized for sample preparation and testing: a laboratory furnace 06-DZ-3BC (Chemland, Stargard, Poland); a WLC 6/A2/C/2/IO precision scale (RADWAG, Radom, Poland), TDR equipment, including a TDR laboratory multimeter (ETest, Lublin, Poland); a 3D-printed TDR sensor (a surface sensor developed as part of the research); and a computer. The latter was used for controlling the TDR multimeter and managing the data.

### 2.7. TDR Multimeter

Measurements were conducted using a TDR multimeter emitting a spike signal with a rise time of 300 ps. The signal is transmitted through the coaxial cable to the sensor, where reflections occur at characteristic points of the propagation line—specifically, at the beginning and end of the sensor. These reflections enable the identification of measurement markers. The TDR multimeter records the time differences between these reflections, which can then be automatically or manually converted into the apparent permittivity value. This permittivity is directly influenced by the moisture content within the material being tested.

### 2.8. Properties of Silicate

Silicate was selected as a building material for the tests. It is composed of quartz sand, quicklime, and water, and is commonly manufactured in the form of silicate bricks, blocks, and hollow bricks. For this research, five silicate samples were prepared with dimensions of 250 mm × 12 mm × 65 mm. The samples were dried to a constant weight and then gradually moistened to achieve a maximum humidity level of 10%. The moisture content of the samples was monitored by weighing them at each stage. The samples were subsequently tested using a non-invasive sensor to collect measurement data. Humidity levels ranging from 0% to 7% and a maximum of 10% were recorded. The estimated apparent density of the material was determined by dividing the mass of the dry material by the sample’s volume. Table 4 presents the parameters of both dry and fully saturated samples.

### 2.9. Description of the Measurement Procedure

This study involved measuring the apparent permittivity of the material under varying moisture content conditions. Measurements were initially conducted on five dry samples, followed by tests on samples with progressively increasing humidity levels until saturation. The experiments were performed in constant temperature (21 °C) and relative air humidity (55%) conditions. The silicate samples measured had dimensions of 250 mm × 12 mm × 65 mm, and a sensor designed for the contact method was used for the tests. Before initiating the main experiments, each sensor channel of the multimeter was calibrated in air and water to identify the locations of the measurement peaks. This calibration was essential for determining the sensor’s dead time (the interval between the reflection on the resistor and the start of the measuring rod) and the measurement step length specific to the multimeter used.

## 3. Results

Through measurements conducted using the prototyped 3D-printed sensor and the TDR meter, raw data were obtained, enabling the identification of certain measurement relationships, which are discussed in detail later in this study.

**Figure 6:**
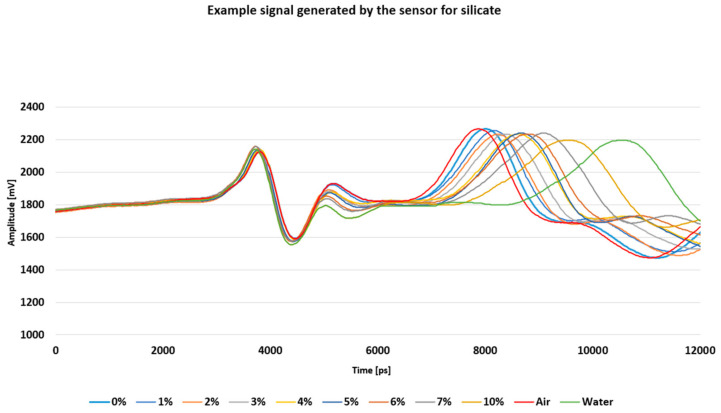
The signal response of the 3D-printed sensor during TDR measurements of silicate samples at varying moisture levels.

Figure 6 presents a representative signal that was generated by the sensor designed for silicate under varying humidity levels. Multiple measurements were conducted to ensure accuracy. Calibration tests validated the performance and reliability of the sensor, which was fabricated using 3D printing technology.

As a results of the measurement data obtained using a 3D sensor and a TDR meter, it was possible to determine the apparent permittivity of silicate at various moisture levels. The dielectric permittivity of a material is determined by the following formula:

(1) $$\varepsilon \;{=\left(\frac{c}{V}\right)}^{2}$$

where *ε* is the dielectric permittivity of the tested material [-], *c* is the speed of light in a vacuum [3 × 10^8^ m/s], and *V* is propagation speed of the electromagnetic pulse along the measuring rods [m/s] [14].

Table 5 presents the results of apparent permittivity for the tested silicates at different moisture levels.

**Figure 7:**
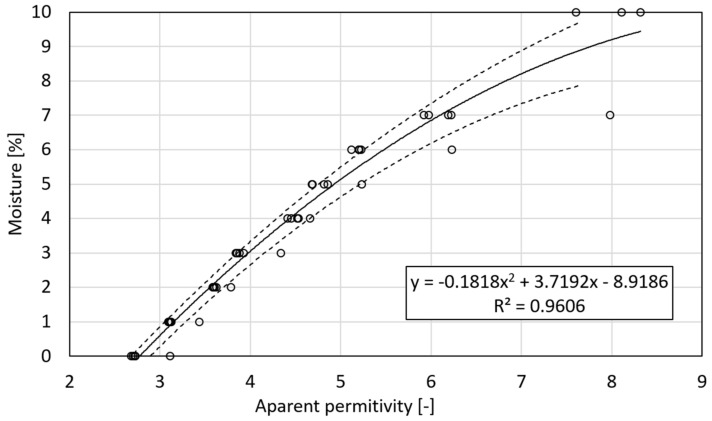
The relationship between moisture and apparent permittivity for the tested silicate.

A model was prepared for the measurement data obtained. The dependence of moisture and apparent permittivity for the tested silicate along with the designated confidence bands is shown in Figure 7.

**Figure 8:**
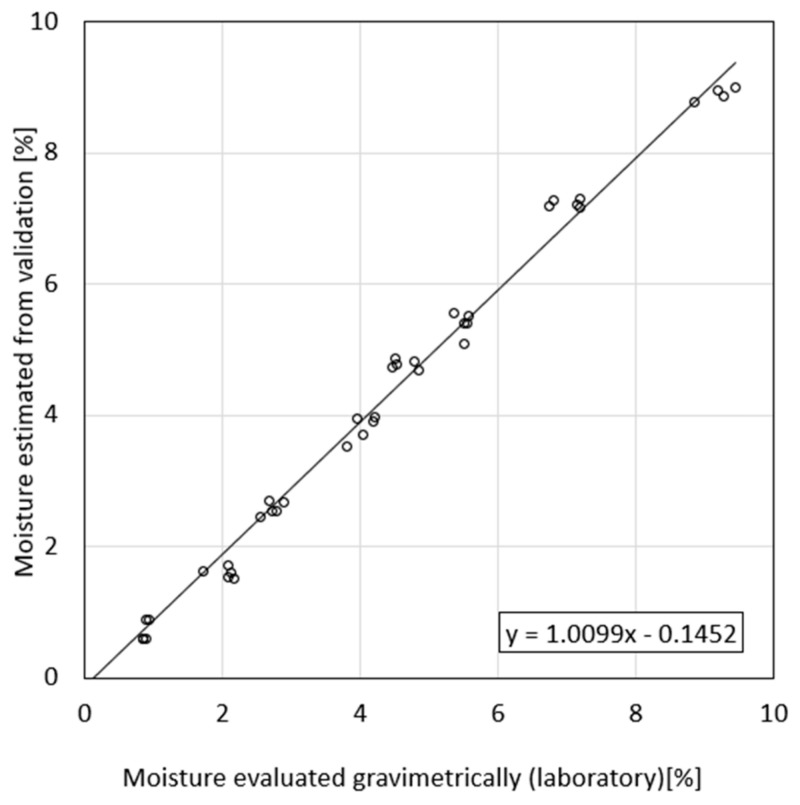
Comparison of estimated moisture content obtained from calibration and moisture evaluated gravimetrically (laboratory).

Figure 8 presents the dependence between material moisture, as estimated in laboratory conditions, and the apparent permittivity, as measured using the TDR equipment. Based on this relation, a regression formula was derived, which in turn served as a calibration model of the TDR sensor. This formula is the third-grade polynomial function and precisely fits to the data obtained because the coefficient of determination equals 0.961, which is close to one.

Linear dependence between the moisture values achieved in laboratory sample preparation and evaluation using the sensor prototype is presented in Figure 8. The results show the good fit between the data obtained and model derived. The slope coefficient of this formula equals 1.0099 and is close to 1 and the y-intercept equals 0.1452 and is close to zero.

Using the classical analytical method, the RMSE for the obtained data was calculated to be 0.70.

### Data Analysis Method

Raw data were collected in the form of a sequence of voltage values as a function of time. The reflections observed are a result of wire discontinuities caused by the sensor’s structure, including the resistor (input peak) and the sensor’s termination (output peak). Based on this, permittivity is determined for calibration using traditional methods, which involves constructing deterministic models. In the case of machine learning approaches, the raw signal, represented as a series of voltage values, is directly analyzed. To facilitate this, a data matrix was constructed, where individual vectors corresponded to voltage values, and the target data consisted of the humidity levels determined through laboratory measurements. The measurement results obtained were imported into MATLAB for analysis using machine learning techniques. The Regression Learner tool was employed, which offers a wide range of algorithms, including linear regression, decision trees, random forests, gradient boosting models, and SVM regression. This tool enables users to compare different models to identify the best fit for their data and facilitates training using various mathematical models. To evaluate the root-mean-square error (RMSE), the data were implemented, and two models were selected: Gaussian Process Regression (GPR) and Support Vector Machine (SVM). The GPR model is an advanced statistical approach used for data modeling and forecasting. It is based on the concept of stochastic processes, assuming that any set of data points can be described by a multivariate Gaussian distribution. This methodology not only provides predicted values, but also gives an estimation of their associated uncertainties. The SVM model, or the Support Vector Machine model, is a widely used machine learning method, particularly for classification and regression tasks. It excels in handling complex datasets and identifying patterns within them. GPR and SVM methods were used to analyze the results. This choice was made due to the fact that the processed raw TDR signal presented a series of voltage changes over time, with only one value recorded for each measurement. The SVM method, although it worked better in the case of multidimensional data, did not fully use its capabilities for the analyzed signal. However, Gaussian Process Regression (GPR) is characterized by greater flexibility and universality when working with one-dimensional data, which allows for the maximum accuracy of readings [54,55]. The results obtained using a surface sensor with a 3D-printed housing were analyzed through machine learning. This was initially performed using Gaussian Process Regression (GPR) and subsequently conducted with Support Vector Machine (SVM). The machine learning outcomes for data acquired via the TDR meter are summarized in Table 6.

**Figure 9:**
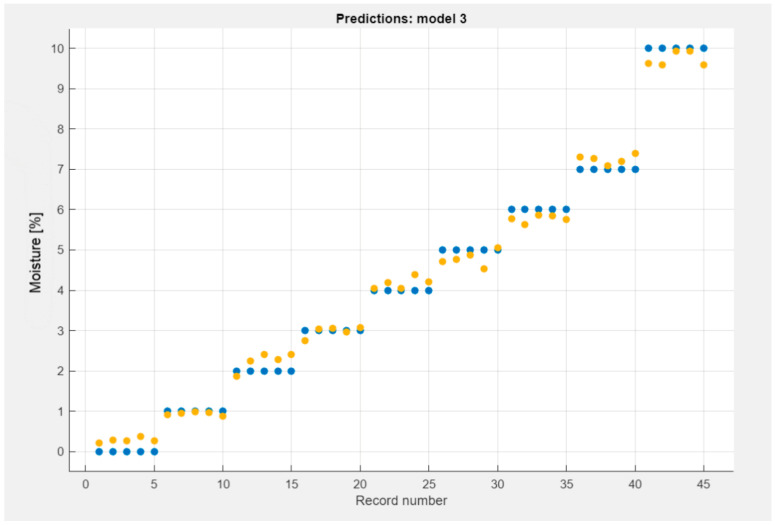
SVM prediction model obtained from the analysis of TDR multimeter data.

**Figure 10:**
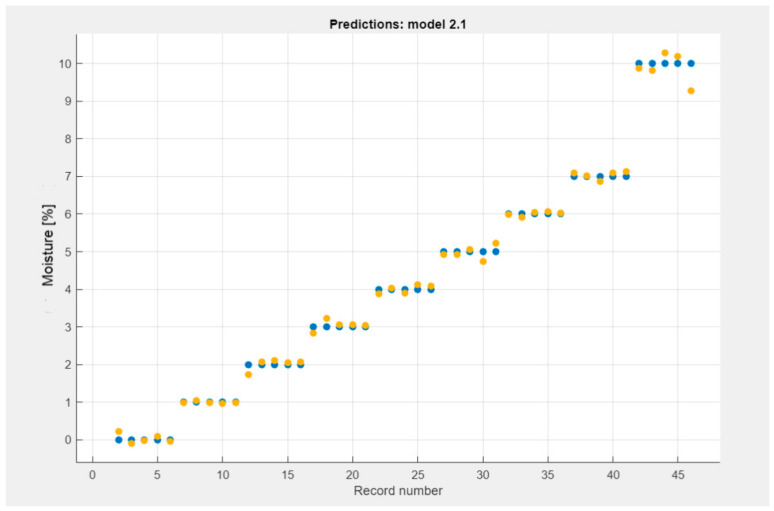
GPR predictions model obtained from the analysis of TDR multimeter data.

Figure 9 and Figure 10 presented the decomposition of the SVM and GPR models.

The predicted model is presented in Figure 9 and Figure 10. Data obtained from machine learning are marked in yellow and raw data obtained from measurements are marked in blue.

## 4. Discussion

The application of 3D printing technology to test materials using a TDR meter is innovative. The results obtained enable detailed analysis through machine learning models such as Support Vector Machine (SVM) and Gaussian Process Regression (GPR). While both methods address regression and classification problems, they differ in operation, assumptions, and applications. SVM training yields a decision function (for classification) or a regression function (for regression) that determines the class assignment or output value. In contrast, the GPR model provides a full probability distribution for each predicted value, offering not only an average prediction but also a range of values representing error and uncertainty. This allows for the calculation of confidence intervals for predicted values. In terms of data training time, the SVM model (7.0181 s) demonstrated significantly faster learning of data obtained via TDR. However, the GPR model (26.751 s) produced better results despite requiring a substantially longer training time for the same dataset. Research by various scientists highlights the extensive applications of TDR technology, which has proven highly effective in numerous fields. These applications include monitoring soil moisture and salinity, detecting infrastructure damage, monitoring geotechnical processes, and testing the environmental parameters of building materials. Research using other sensors, i.e., invasive ones, was carried out by, among others, Suchorab et al. [38], Blonquist Jr, Scot be Jones [56], and Hansen and Jan [57]. The method of invasive measurement of building materials was also presented by Fiala [58] and Pavlik [59]. Most of the presented authors of tests performed with invasive sensors primarily undertook measurements of aerated concrete due to the ease of installing an invasive probe in this material. The less dense the aerated concrete, the easier it is to place the measuring rods of the probes in it, but this also destroys the sample. In a study by Paśnikowska et al. [60], the comparison of invasive and non-invasive probes was undertaken. However, the probers were made of other different materials, i.e., black polyoxymethylene, which is not as easily available as PLA. The authors emphasized that TDR sensors provide very good responses that are similar to measurements made using traditional invasive sensors. For example, the electrical permeability for 600 cellular concretes, determined from the data obtained using an invasive probe for a saturated sample, i.e., 20%, had an apparent permittivity of about 5.5 [-], and the measurements made using the mentioned non-invasive sensor on the same 20% sample allowed us to determine apparent permittivity at an average level of about 5.3 [-]. These data are very similar, so it can be concluded that surface sensors can also be used to determine measurement data.

The results indicate that sensors fabricated using 3D printing technology are comparable in performance to the conventional sensors described by Suchorab, Pavlík, and Černý [60]. Materials with varying porosities exhibit distinct characteristics, enabling their quality to be evaluated using 3D-printed sensors. Increased material porosity results in a prolonged transit time of the electric pulse due to the higher number of voids (pores), which can lead to signal reflections. The silicate material analyzed in this study demonstrates lower porosity compared to commonly used cellular concrete.

## 5. Conclusions

Categorized as non-invasive devices, TDR surface sensors, including the sensor employed in this study, demonstrate that TDR technology can be effectively applied to investigate the moisture content of porous construction materials such as silicate. These non-invasive methods preserve the integrity of the tested materials, offering a significant advantage in applications requiring non-destructive testing.

The integration of 3D printing technology into the fabrication of surface sensors enhances the flexibility and adaptability of this method, enabling the customization of sensors to accommodate various material shapes. At each stage of the design process, appropriately sized sensors can be manufactured to optimize compatibility with the tested material. The analysis of existing research, coupled with results from this study, confirms that TDR surface sensors can produce mapping results comparable to those obtained with traditional invasive sensors. This characteristic makes the method particularly valuable in conservation-sensitive areas, where material preservation is critical. Moreover, if the sensor housing is damaged, a replacement can be quickly fabricated using 3D printing technology.

Prototyping sensors with this approach has proven highly effective in environmental parameter studies. Silicate serves as an excellent material for environmental testing, and is capable of absorbing moisture up to 10% of its mass. However, its drying time is significantly longer than that of materials such as aerated concrete. Data collected using the surface sensor and TDR meter can be incorporated into machine learning models to achieve highly accurate results. Machine learning, widely adopted to analyze TDR-generated research data, further validates the method’s precision.

The highest apparent permittivity reached a value of 8.32 [-] for a sample maximally saturated with 10%, whereas at 1% absorption, the apparent permittivity was 2.62 [-].

In this study, the test results yielded an RMSE of 0.15 for the GPR model and one of 0.25 for the SVM model, indicating that GPR provides a more accurate representation of the measurements and is better suited to detailed analysis. In contrast, traditional data analysis methods produced an RMSE of 0.70, underscoring the superiority of machine learning in this context.

The measurements also enabled the determination of the apparent permittivity of silicate, averaging 2.7 [-] for dry samples (based on five measurements) and 7.65 [-] for samples at maximum absorbability.

The presented research method is an indirect method, and its greatest limitation is the influence of foreign sensors on the reading of the sensor signal. These include temperature, air humidity, and the presence of salt ions. Another limitation is the sensor range, which in the case of the analyzed structures ranges from 3 to 4 cm, depending on the spacing of the measuring rods.

The tested material from which the sensor was made is stable in laboratory conditions because the melting point of the material (PLA) is 180 degrees Celsius, which is much higher than the operating temperature of around 20 degrees Celsius. This ensures that the measurement accuracy of the method determined in the experiment is maintained.

In order to increase the functionality of the sensor, it should also be tested for porous media with different densities, including materials such as aerated concrete, ceramic, or clinker bricks.

The testing process using a surface sensor is faster than that with an invasive sensor. To sum up, the use of 3D printing in the preparation of non-invasive surface sensors may be an innovative step in further research on the environmental parameters of building materials. In the future, tests should also be carried out on probes of other dimensions and using other filaments, e.g., ABS, to check the effectiveness of the measurement method.

Research conducted on the prototype probe highlights the importance of considering damping effects on measurement accuracy in future studies. The development of regression models is recommended to further improve measurement precision. Additionally, research on 3D-printed probes should address the influence of external ions, and future efforts should examine the impact of probe geometry on the accuracy of the measurement method.

## Figures and Tables

**Table 1:** The parameters of the 3D printer used for the sensor prototype (manufacturer’s data).

Parameters of the 3D Printer	
Working area	220 mm × 220 mm × 250 mm
Material diameter	1.75 mm
Diameter of the print head	0.4 mm
Number of printheads	1 filament
Device dimensions	475 mm × 470 mm × 620 mm
Maximum table temperature:	110 °C
Maximum head temperature	250 °C
Printing accuracy	0.1 mm
Print speed	50 mm/s
The layer’s height	0.28 mm
Fill pattern	Lines
Infill percentages	100%

**Table 2:** The parameters of the PLA (Spectrum manufacturer’s data).

Material	PLA
Diameter of material	1.75 mm
Print temperature	185 °C to 215 °C
Color	Deep Black
Heat distortion temperature	55 °C
Flexural modulus	3.8 [MPa]
Flexural strength	83 [MPa]
Notched Izod impact	16 J/m
Specific gravity	1.24 g/cm^3^
Tensile strength at break	53 [MPa]
Electrical conductivity	<10^−12^ S/m

**Table 3:** Checking the absorbability of the PLA used.

Deep Black PLA in pH 7 Aqueous Solution	Weight			
	1st Day [g]	5th Day [g]	13th Day [g]	1st Month [g]
1	18	18	18	18
2	18	18	18	18
3	18	18	18	18
4	18	18	18	18
5	17	17	17	17

**Table 4:** Parameters of silicate samples (PPMB Niemce S.A.).

Number of Samples	Density	0% [g]	10% [g]
1	3.63	3613.0	3990.4
2	3.63	3633.0	3998
3	3.71	3700.0	4081.5
4	3.63	3610.4	3988.1
5	3.29	3629	3616

**Table 5:** Apparent permittivity values of the tested silicates.

Apparent Permittivity *ε*_1–5_ [-]					
Moisture [%]	1	2	3	4	5
0	2.68	2.68	2.72	2.70	2.73
1	2.72	3.11	3.10	3.09	3.12
2	3.11	3.44	3.59	3.60	3.58
3	3.62	3.78	3.88	3.84	3.85
4	3.93	4.34	4.45	4.41	4.54
5	4.52	4.66	4.69	4.82	4.68
6	4.85	5.23	5.12	5.23	5.20
7	5.21	6.23	6.19	5.92	5.98
10	6.22	7.98	7.61	8.11	8.32

**Table 6:** The obtained results of machine learning of data obtained using a TDR meter (confidence intervals 95%).

Training Results		
Model	GPR	SVM
RMSE (validation)	0.15443	0.24706
R^2^	1.00	0.99
MSE (validation)	0.023849	0.061039
MAE (validation)	0.10851	0.2091
Prediction speed	~50 obs/s	~68 obs/s
Training time	26.751 s	7.0181 s
Model size (compact)	~916 kB	~441 kB

## Data Availability

The original contributions presented in the study are included in the article, further inquiries can be directed to the corresponding authors.
